# Using patient-reported symptoms of dyspnea for screening reduced respiratory function in patients with motor neuron diseases

**DOI:** 10.1007/s00415-020-10003-5

**Published:** 2020-06-23

**Authors:** Jochem Helleman, Esther T. Kruitwagen-van Reenen, J. Bakers, Willeke J. Kruithof, Annerieke C. van Groenestijn, Rineke J. H. Jaspers Focks, Arthur de Grund, Leonard H. van den Berg, Johanna M. A. Visser-Meily, Anita Beelen

**Affiliations:** 1 Department of Rehabilitation, Physical Therapy Science and Sports, University Medical Center Utrecht, Heidelberglaan 100, 3584 CX Utrecht, The Netherlands; 2 Center of Excellence for Rehabilitation Medicine, UMC Utrecht Brain Center, University Medical Center Utrecht, and De Hoogstraat Rehabilitation, Utrecht, The Netherlands; 3 Department of Neurology, UMC Utrecht Brain Center, University Medical Center Utrecht, Utrecht, The Netherlands; 4 Department of Rehabilitation, Amsterdam UMC, University of Amsterdam, Amsterdam Movement Sciences, Amsterdam, The Netherlands; 5 Roessingh, Center for Rehabilitation, Enschede, The Netherlands; 6 Basalt, Center for Rehabilitation, Den Haag, The Netherlands

**Keywords:** Motor neuron disease, Amyotrophic lateral sclerosis, Dyspnea, Vital capacity, Respiratory function, Patient-reported outcome measure

## Abstract

**Background:**

Poor monitoring of respiratory function may lead to late initiation of non-invasive ventilation (NIV) in patients with motor neuron diseases (MND). Monitoring could be improved by remotely assessing hypoventilation symptoms between clinic visits. We aimed to determine which patient-reported hypoventilation symptoms are best for screening reduced respiratory function in patients with MND, and compared them to the respiratory domain of the amyotrophic lateral sclerosis functional rating scale (ALSFRS-R).

**Methods:**

This prospective multi-center study included 100 patients with MND, who were able to perform a supine vital capacity test. Reduced respiratory function was defined as a predicted supine vital capacity ≤ 80%. We developed a 14-item hypoventilation symptom questionnaire (HYSQ) based on guidelines, expert opinion and think-aloud interviews with patients. Symptoms of the HYSQ were related to dyspnea, sleep quality, sleepiness/fatigue and pneumonia. The diagnostic performances of these symptoms and the ALSFRS-R respiratory domain were determined from the receiver operating characteristic (ROC) curves, area under the curve (AUC), sensitivity, specificity, predictive values and accuracy.

**Results:**

Dyspnea-related symptoms (dyspnea while eating/talking, while lying flat and during light activity) were combined into the MND Dyspnea Scale (MND-DS). ROC curves showed that the MND-DS had the best diagnostic performance, with the highest AUC = 0.72, sensitivity = 75% and accuracy = 71%. Sleep-quality symptoms, sleepiness/fatigue-related symptoms and the ALSFRS-R respiratory domain showed weak diagnostic performance.

**Conclusion:**

The diagnostic performance of the MND-DS was better than the respiratory domain of the ALSFRS-R for screening reduced respiratory function in patients with MND, and is, therefore, the preferred method for (remotely) monitoring respiratory function.

**Electronic supplementary material:**

The online version of this article (10.1007/s00415-020-10003-5) contains supplementary material, which is available to authorized users.

## Introduction

Motor neuron diseases (MND) are rapidly progressive neurodegenerative diseases, which include amyotrophic lateral sclerosis (ALS), progressive muscular atrophy (PMA) and primary lateral sclerosis (PLS). The main cause of death is respiratory failure due to diaphragm weakness [1]. One of the first signs of diaphragm weakness is nocturnal hypoventilation. Prolonged hypoventilation leads to hypercapnia, which causes clinical symptoms, such as sleep disturbances and daytime fatigue [2]. These symptoms may negatively affect patients’ quality of life.

Non-invasive ventilation (NIV) is the most effective intervention for improving quality of life and relieving symptoms in patients with MND (2). The effect of NIV on the rate of respiratory decline and survival has been shown to be associated with the timing of NIV initiation [3, 4]. The guidelines for MND specify a number of criteria for the timing of NIV initiation, based on pulmonary function tests, blood gas analysis and respiratory symptoms [5–7]. Despite these guidelines, NIV is often initiated late, which could lead to reduced compliance and worse survival [8]. Literature suggests that poor monitoring of respiratory function as a result of lacking pulmonary function tests, may be one of the causes of late NIV initiation [9–13].

One way to improve the monitoring of respiratory function is by remotely monitoring respiratory symptoms that are indicative of a reduced pulmonary function test score. This approach enables more frequent assessments of respiratory function compared to usual care, may facilitate early detection of respiratory dysfunction and allows patients to stay at home, saving travel time and costs. Unlike performing pulmonary function tests, the remote assessment of respiratory symptoms is simple, not requiring a medical device or skill to complete.

Currently, the respiratory domain of the revised amyotrophic lateral sclerosis functional rating scale (ALSFRS-R) is commonly used for assessing respiratory symptoms in patients with MND. However, there has been some criticism about the screening value of this domain [14, 15]. For this reason, it would be valuable to know which symptoms are better than the respiratory domain of the ALSFRS-R for screening a reduced pulmonary function test score. In clinical care, the vital capacity (VC) test is the most used pulmonary function test for assessing respiratory function in patients with MND. Knowing which symptoms are best for screening a reduced VC will help healthcare professionals to remotely identify those who may need to be referred to a pulmonologist for comprehensive assessment.

We, therefore, developed a hypoventilation symptom questionnaire (HYSQ), based on guidelines, expert opinion and think-aloud interviews with patients, and compared the diagnostic performance of the HYSQ with the respiratory domain of the ALSFRS-R.

## Methods

### Hypoventilation symptom questionnaire

The patient-reported hypoventilation symptom questionnaire (HYSQ) was developed to standardize the assessment of hypoventilation symptoms. Recent literature has provided evidence that patient-reported outcome measures are feasible and of added value in routine ALS care [16]. A preliminary questionnaire of 19 items was created based on literature, expert opinion and guidelines for management of MND [5, 6]. It assessed the extent to which patients experienced the symptoms of hypoventilation. Items could be scored from 0 (not at all) to 4 (to a great extent). Think-aloud interviews were conducted, in which patients verbalize their thoughts as they read and complete the questionnaire, to investigate whether they were able to understand the items and whether they used correct reasoning when answering the items. A total of 10 think-aloud interviews were conducted with ALS patients. After completing these interviews, items were adjusted linguistically or removed when patients did not fully understand them. The final version of the HYSQ consisted of 14 items: Disturbed sleep, difficulty returning to sleep, nightmares, night sweats, waking up tired, morning headache, daytime sleepiness, fatigue, concentration problems, pneumonia, dyspnea while seated, dyspnea while eating/talking, dyspnea while supine and dyspnea during light activity (Table 1). The full questionnaire can be found in Online Resource 1.

**Table 1:** The hypoventilation symptom questionnaire and the diagnostic performance of individual items

Hypoventilation Symptom Questionnaire	Diagnostic performance (ROC analysis)	
	AUC	*p *value^a^
1. At night I wake up often	0.58	0.18
2. When I wake up at night, it takes a long time before I fall asleep again	0.50	0.99
3. At night I have nightmares	0.54	0.46
4. I wake up at night/in the morning drenched in sweat	0.46	0.54
5. I feel tired when I wake up in the morning	0.50	0.99
6. I experience headaches after I wake up in the morning	0.57	0.27
7. I find it difficult to stay awake during the day (e.g. while watching TV or reading a book)	0.59	0.15
8. I experience fatigue during the day	0.50	0.99
9. I have difficulties concentrating (e.g. when watching TV or reading a book)	0.49	0.83
10. I suffer from pneumonia (i.e. excessive coughing and mucus in my throat)	0.65	< 0.05
11. I feel short of breath when sitting still	0.62	< 0.05
12. I feel short of breath when talking or eating	0.66	< 0.01
13. I feel short of breath when I lie flat on my back^b^	0.66	< 0.01
14. I feel short of breath during light activities (e.g. walking, washing or getting dressed)	0.72	< 0.01

*ROC* receiver operating characteristic, *AUC* area under the curve. ^a^Indicates whether the AUC is significantly higher than 0.5 (= chance). ^b^Also known as orthopnea

### Study design and population

This prospective multi-center study aimed to include 100 consecutive patients with ALS, PMA or PLS, aged 18 and over. Patients were excluded from the study if they were receiving NIV or tracheostomy ventilation, unable to understand the questionnaire due to cognitive dysfunction or when a correct performance of the pulmonary function test was not possible due to bulbar impairment. Ethics approval was obtained prior to the start of the study and patients gave informed consent before participating.

### Setting and procedure

A total of six multidisciplinary ALS clinics in the Netherlands participated in the current study, three of which were rehabilitation centers and three university medical centers. During a regular visit to a multidisciplinary clinic, between August 2018 and November 2019, patients were invited by a rehabilitation physician or physical therapist to take part in the study. Patients were asked to perform a pulmonary function test three times and fill in the HYSQ and the ALSFRS-R. The ALSFRS-R is a validated questionnaire that indicates the level of functional impairment in four domains of functioning: bulbar function, fine and gross motor skills, and respiratory function [17]. Each domain consists of 3 items that are scored from 0 (fully impaired) to 4 (not impaired), resulting in a total score between 0 (worst) and 48 (best).

#### Pulmonary function test

The pulmonary function test used in the current study was the forced vital capacity (FVC) test in supine position. It has been shown that this test can predict diaphragm weakness and survival better than an upright FVC or the difference between the upright and supine FVC [18–20]. In addition, the supine FVC has been highly correlated to the trans-diaphragmatic pressure, which is the ‘gold standard’ for assessing diaphragmatic weakness [21]. Patients who were not able to perform the supine FVC correctly (e.g. due to air leakage or orthopnea), were allowed to perform a slow vital capacity (SVC) test in supine position. Literature has shown that the results of the SVC and FVC are very similar and interchangeable [22, 23]. We, therefore, report the supine VC.

#### Test–retest HYSQ

We aimed to include 50 patients in the test–retest analysis. One week after the baseline assessment, the patients were sent an e-mail with a digital link to an online version of the HYSQ on a secure survey website. Patients had one week to fill in the online questionnaire; if they exceeded this time period, the retest was invalid and not used in the reliability analysis.

### Analyses

The highest value of three supine VC test attempts was converted to a percentage of the predicted VC, using age, height and ethnicity, according to the reference values from the Global Lung Function Initiative 2012. The threshold for a reduced respiratory function was ≤ 80% of the predicted supine VC [6].

In current clinical practice, items 10 (dyspnea), 11 (orthopnea) and 12 (respiratory insufficiency) of the ALSFRS-R are used to assess respiratory function in patients with MND. In the present study, we excluded item 12 from the analysis as it assesses whether patients use NIV, which was an exclusion criterion. We report the ALSFRS-R_10,11_.

Relative operating characteristics (ROC) curves and the area under the curve (AUC) were obtained for all individual HYSQ items. Items with an AUC ≥ 0.6 and asymptotic significance (*p* < 0.05) were combined into an HYSQ sum score. An exploratory factor analysis was performed with a varimax rotation to determine the factor structure and constructs of the HYSQ. Items were considered to contribute to a factor with a factor loading ≥ 0.5.

ROC curves and the AUC were obtained for the HYSQ sum score, HYSQ factors and ALSFRS-R_10,11_. The ROC curves were assessed to determine the optimal cut-off score for correct identifications of reduced respiratory function (as a dichotomous outcome). Sensitivity, specificity, positive predictive value (PPV), negative predictive value (NPV) and accuracy (true-positive rate + true negative rate) were calculated. A finding was considered a true-positive, when a patient experienced symptoms based on the optimal cut-off score, with a supine VC ≤ 80%, and a finding was considered a true negative, when a patient did not experience symptoms based on the cut-off score, with a supine VC > 80%. A correlation analysis was performed to determine the strength of the relationship between the symptoms (HYSQ and ALSFRS-R_10,11_) and respiratory function.

The test–retest reliability of the HYSQ sum score and HYSQ factors were determined using the Intraclass Correlation Coefficient (ICC) and the absolute reliability was determined using the minimal detectable change (MDC). The MDC with a confidence interval of 95% was calculated with the standard error of the mean (SEM) using the formula: MDC = SEM × √2 × 1.96. All analyses were performed with SPSS 25 software.

## Results

A total of 100 patients were included in the current study. Their average age was 64, 63% were male, 63% were diagnosed with ALS, 81% had spinal onset and the mean ALSFRS-R score was 36. One patient did not perform a supine VC and was, therefore, excluded from the analysis. Nine patients had missing ALSFRS-R data. All patient characteristics are presented in Table 2.

**Table 2:** Patient characteristics

Characteristic	Patients (*N* = 100)	*N*
Gender (male), *n* (%)	63 (63.0)	100
Age (years), *mean (SD)*	63.8 (10.4)	100
Ethnicity, *n* (%)		100
Caucasian	96 (96.0)	
Asian	2 (2.0)	
Other/mixed	2 (2.0)	
Current smoker, *n* (%)	20 (20.2)	99
Diagnosis, *n* (%)		99
ALS	62 (62.6)	
PMA	23 (23.2)	
PLS	14 (14.1)	
Site of onset, *n* (%)		99
Bulbar	19 (19.2)	
Spinal	80 (80.8)	
Comorbidities, *n* (%)		100
COPD	6 (6.0)	
Apnea	1 (1.0)	
None	93 (93.0)	
Respiratory function (% of predicted supine VC), *mean (SD)*	72.4 (22.7)	99
Reduced respiratory function (≤ 80% predicted supine VC), *n* (%)	56 (56.6)	99
Disease duration from diagnosis (months), *median (IQR)*	14.3 (6.2–29.2)	97
Diagnostic delay (months), *median (IQR)*	12.5 (6.5–26.0)	97
ALSFRS-R, *mean (SD)*	36.0 (7.0)	91
ALSFRS-R (respiratory domain),* mean (SD)*	11.2 (1.5)	91
ALSFRS-R_10,11_, *mean (SD)*	7.2 (1.3)	91

*ALS *amyotrophic lateral sclerosis, *PMA *progressive muscular atrophy, *PLS *primary lateral sclerosis, *COPD *chronic obstructive pulmonary disease, *VC *vital capacity, *SD *standard deviation, *IQR *interquartile range, *ALSFRS-R *revised ALS functional rating scale, *ALSFRS-R*_10,11_ ALSFRS-R items 10 and 11

**Fig. 1:**
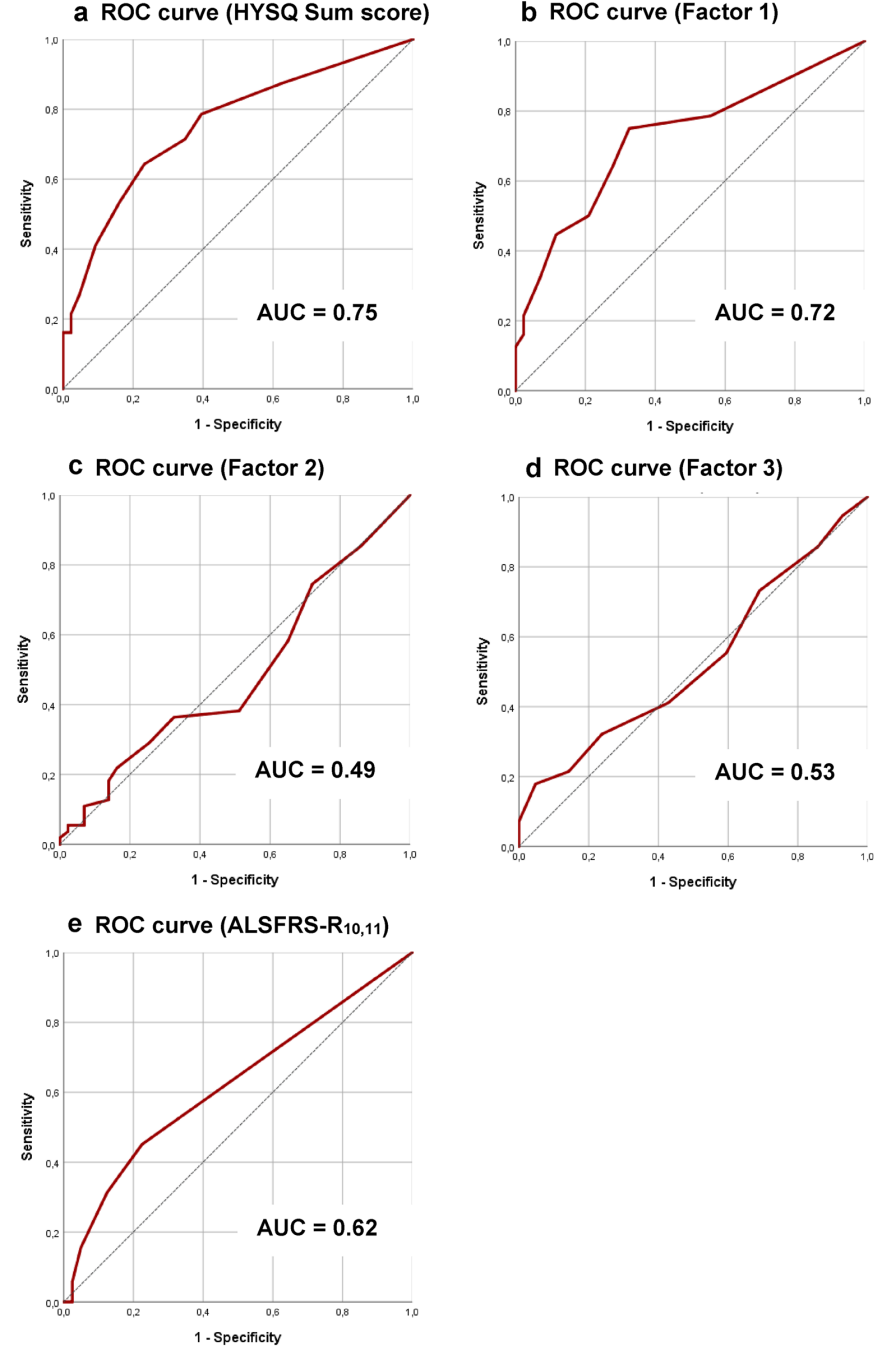
The ROC curves for predicting reduced respiratory function as a dichotomous outcome. HYSQ sum score = items 10 to 14, Factor 1 = items 3, 6, 11 to 14, Factor 2 = items 1, 2, 4 and 5, Factor 3 = items 7 to 9, ALSFRS-R_10,11_ = items 10 and 11 of the revised amyotrophic lateral sclerosis functional rating scale, *ROC* receiver operating characteristic, *AUC* area under the curve

The AUC of all individual HYSQ items are presented in Table 1. Items 10 to 14 proved to have the best individual diagnostic performance with an AUC > 0.6 and asymptotic significance (*p* < 0.05); they were combined into an HYSQ sum score. An exploratory factor analysis identified three factors: factor 1 was related to dyspnea, factor 2 to sleep quality, and factor 3 to sleepiness/fatigue. Pneumonia was the only symptom that did not correlate well with any of these three factors (Table 3). The ROC curve and AUC of the HYSQ sum score, HYSQ factors and the ALSFRS-R_10,11_ are presented in Fig. 1. The largest AUCs, were observed in the HYSQ sum score (0.75) and factor 1 (0.72), followed by the ALSFRS-R_10,11_ (0.62). The optimal cut-off score for correct identification of a reduced respiratory function was ≥ 2 for factor 1, factor 2 and the HYSQ sum score, ≥ 3 for factor 3, and ≤ 7 for the ALSFRS-R_10,11._ The sensitivity, specificity, PPV, NPV and accuracy are shown in Table 4. The HYSQ sum score and factor 1 showed the best diagnostic performance, with an accuracy of 71% (true-positive rate = 42%) and 72% (true-positive rate = 43%), respectively. The HYSQ sum score and factor 1 misclassified 17% and 14% of patients as having a reduced respiratory function (false positives), and misclassified 12% and 14% of patients as having normal respiratory function (false negatives), respectively.

**Table 3:** Factor analysis results

HYSQ symptom	Factor loading
**Factor 1**	
3. Nightmares	0.66
6. Morning headache	0.75
11. Dyspnea while seated	0.81
12. Dyspnea while talking/eating	0.66
13. Dyspnea while lying flat^a^	0.90
14. Dyspnea during light activity	0.65
**Factor 2**	
1. Restless sleep	0.81
2. Difficulty returning to sleep	0.89
4. Nightsweats	0.54
5. Waking up unrefreshed	0.74
**Factor 3**	
7. Daytime sleepiness	0.77
8. Fatigue	0.61
9. Concentration problems	0.77
None	
10. Pneumonia	–

Three factors were identified with an eigenvalue greater than 1. The factor loading indicates the strength of the relationship between the HYSQ symptom and the identified factors. ^a^Also known as orthopnea

**Table 4:** Diagnostic performance and reliability

Statistic	HYSQ sum score	Factor 1	Factor 2	Factor 3	ALSFRS-R_10,11_	Revised HYSQ sum score	Revised factor 1-a	Revised factor 1-b (MND-DS)
Sensitivity (%)	72.1	75.0	56.9	58.6	71.9	72.1	75.0	74.5
Specificity (%)	68.4	67.4	46.2	46.4	52.5	68.4	67.4	65.9
PPV (%)	78.6	75.0	74.5	73.2	45.1	78.6	75.0	73.2
NPV (%)	60.5	67.4	27.9	31.0	77.5	60.5	67.4	67.4
Accuracy (%)	70.7	71.7	53.5	54.5	54.5	70.7	71.7	70.7
Correlation (*r*)	− 0.51*	− 0.50*	− 0.12	− 0.08	0.30*	− 0.50*	− 0.50*	− 0.50*
ICC	0.85	0.89	0.88	0.85	n.a	0.85	0.89	0.88
MDC	1.23	1.32	1.04	0.75	n.a	1.00	1.08	0.81

HYSQ sum score = items 10 to 14, Factor 1 = items 3, 6, 11 to 14, Factor 2 = items 1, 2, 4 and 5, Factor 3 = items 7 to 9, Revised HYSQ sum score = items 10, 12 to 14, Revised factor 1-a = items 3, 6, 12 to 14, Revised factor 1-b = items 12 to 14. *HYSQ* hypoventilation symptom questionnaire, *MND-DS* Motor Neuron Disease Dyspnea Scale, *PPV* positive predictive value, *NPV* negative predictive value, *Accuracy* true-positive rate + true negative rate, *ICC* intraclass correlation coefficient, *MDC* minimal detectable change, *n.a.* not applicable. *p = 0.05

Further analysis of the HYSQ sum score and factor 1 showed that item 11 (dyspnea while seated) was only present when other types of dyspnea were already more severe. For this reason we assumed that *dyspnea while seated* was not of added value for the early screening of respiratory function. Accordingly, we obtained the ROC curve of the revised HYSQ sum score and revised factor 1-a (item 11 removed), and observed equal AUCs, and no changes in sensitivity, specificity, PPV, NPV or accuracy. When we looked further into revised factor 1-a, items 3 (nightmares) and 6 (morning headache) did not seem to fit the construct of dyspnea well, and in addition, they had the lowest AUCs. For this reason, we evaluated their contribution to the diagnostic performance of the revised factor 1-a. We found that the removal of *nightmares* did not lead to any changes in any of the diagnostic performance parameters. The removal of *morning headache* resulted in one true positive changing to a false negative, therefore slightly reducing the accuracy from 72 to 71%. However, due to the lack of contribution of items 3 and 6 to the diagnostic performance, we removed these items from the revised factor 1-a. The diagnostic performance parameters of the revised factor 1-b (items 3, 6 and 11 removed) and revised HYSQ sum score are presented in Table 4 with the optimal cut-off score of ≥ 2. With higher cut-off scores (3 to 6), the sensitivity of the revised factor 1-b showed the largest increase compared to the revised HYSQ sum score and (unrevised) factor 1 (Table 5).

**Table 5:** Sensitivity with higher symptom cut-off scores

Variables	Cut-off scores				
	≥ 2*	≥ 3	≥ 4	≥ 5	≥ 6
HYSQ sum score	72.1	72.7	78.3	81.1	85.2
Factor 1	75.0	75.0	75.7	83.3	85.7
Revised HYSQ sum score	72.1	71.7	79.5	83.3	88.0
Revised factor 1-b (MND-DS)	74.5	75.6	81.3	90.0	93.8

*HYSQ* hypoventilation symptom questionnaire, *MND-DS* Motor Neuron Disease Dyspnea Scale. *The cut-off score for an optimal area under the curve

The revised HYSQ sum score (*r* = − 0.50, *p* < 0.001), revised factor 1-b (*r* = − 0.50, *p* < 0.001), and the ALSFRS-R_10,11_ (*r* = 0.30, *p* = 0.004) were significantly correlated to respiratory function, but factor 2 (*r* = − 0.12, *p* = 0.26) and factor 3 (*r* = − 0.08, *p* = 0.42) were not. The retest was completed by 48 patients, on average 9 days after the baseline assessment. The test–retest reliability and absolute reliability were shown to be good in all variables, with high ICC values and low MDC values (Table 3). The MDC values of the HYSQ sum score and factor 1 improved after they were revised.

Based on our results, we concluded that revised factor 1-b had the best overall diagnostic performance and reliability. For this reason the symptoms of revised factor 1-b (dyspnea while eating/talking, dyspnea while lying flat and dyspnea during light activity) were combined into the Motor Neuron Disease Dyspnea Scale (MND-DS) (see Online Resource 2). Each item of this scale can be scored from 0 to 4, resulting in a possible total score between 0 (no dyspnea) and 12 (severe dyspnea), with an optimal cut-off-score of ≥ 2 and 75% sensitivity.

## Discussion

We have developed a patient-reported scale (MND-DS) that, in the majority of cases, is able to identify whether or not patients with MND have reduced respiratory function. The MND-DS proved to have a better diagnostic performance than the respiratory domain of the ALSFRS-R. This suggests that it is preferable to use the new scale for monitoring respiratory function and for referring patients to a pulmonologist for comprehensive assessment. Furthermore, sleep quality-related symptoms and sleepiness/fatigue-related symptoms were weak in identifying reduced respiratory function and had a weak relationship with supine VC. This demonstrates that in the present study these symptoms were less suitable for monitoring respiratory function.

Our findings suggest that of all hypoventilation symptoms, dyspnea (and orthopnea) could best be used for remote monitoring to screen for reduced respiratory function in patients with MND. Another reason for monitoring these symptoms regularly is that they are well-correlated with, and good predictors of, NIV use [24–26]. Correspondingly, studies have shown that most healthcare professionals in Europe and the United States consider the symptoms of dyspnea and orthopnea to be (one of) the most important parameters for prescribing NIV to patients with MND [8, 11, 27, 28]. In these studies, sleep-related symptoms were often also considered important when deciding on prescribing NIV, and in the Netherlands they were used more often than dyspnea and orthopnea when deciding on NIV [28]. Interestingly, our study showed that sleep-related symptoms had low diagnostic performance and a weak relationship with supine VC. A reason for this finding could be that the supine VC is not an appropriate measure for assessing sleep disordered breathing. Future research, could evaluate the diagnostic performance of sleep-related symptoms with an overnight polysomnography, which is the gold standard for assessing sleep disordered breathing. Another possible reason for the weak relationship is that sleep disturbances may be caused by non-respiratory factors such as muscle cramps, pain, reduced mobility, choking or anxiety [24, 29]. Accordingly, a study has shown that sleep-related symptoms did not correlate with nocturnal abnormalities of blood carbon dioxide and oxygen levels [30]. These findings suggest that it may be better for healthcare professionals to assess dyspnea and orthopnea when trying to identify patients with a reduced respiratory function, and that sleep-related symptoms should be assessed for the detecting sleep disturbances and possibly sleep disordered breathing.

When we look at previous studies that have investigated the relationship between hypoventilation symptoms and respiratory function, we find contrasting results. Jackson and colleagues [20] investigated the relationship between a hypoventilation symptom scale (similar to the HYSQ) and various pulmonary function tests (including supine VC) in a small sample of 13 patients with ALS. They found no significant correlations between any of the pulmonary function tests and the total score of the symptom scale. A possible reason is that they combined all symptoms into one score, which means that weakly correlated items also contributed to the total score. Another study also found no correlation between a validated dyspnea questionnaire and three respiratory measures (including supine VC) in patients with ALS [31]. This 15-item questionnaire covered different aspects of dyspnea, including the emotional burden/distress caused by dyspnea. Several items asked whether dyspnea leads to feelings of isolation, depression and fear. These aspects may vary in individuals with similar levels of respiratory dysfunction, and may, therefore, have a weaker relationship with respiratory function. Furthermore, patients who were on NIV were included in the study, which could have affected the results, since NIV is known to relieve dyspnea-related symptoms.

When using the MND-DS for monitoring, a subgroup of patients will be misclassified as having normal respiratory function (false negative) or as having reduced respiratory function (false positives). Despite the misclassification of false positive patients, these patients were symptomatic with dyspnea and/or orthopnea, which is an important indication for NIV initiation and therefore a valid reason for referral to the multidisciplinary care team or a pulmonologist [26, 27]. The group of false negative patients, however, was identified as asymptomatic, meaning they would not be referred whilst having reduced respiratory function. This shows that a lack of symptoms in patients with MND does not exclude a reduced respiratory function. For this reason, the MND-DS should be used for monitoring between clinic visits, in combination with regular in-clinic pulmonary function testing. To identify all patients with reduced respiratory function through remote monitoring, the assessment of symptoms should be combined with home-based VC testing. A recent study has demonstrated that patients and their caregivers are able to perform a VC test reliably when following live-video instructions in a clinical setting [32]. It is not known, however, whether patients with MND are able to validly and reliably perform VC tests independently at home. For this reason, future studies could compare home-based VC tests performed by patients with MND (and their caregiver) with those performed in-clinic by a healthcare professional.

### Strengths and limitations

The strengths of the present study are the multi-center design and the relatively large cohort of patients with MND. We chose not to use the gold standard (i.e. trans-diaphragmatic pressure) for assessing respiratory function in the present study, as this method is invasive and labor intensive. Instead we used the supine VC, which is highly correlated to trans-diaphragmatic pressure, widely available and feasible in clinical practice. The assessment of the supine VC was performed with validated spirometers by experienced physical therapists or rehabilitation physicians. The assessments were not, however, standardized across centers, which may have caused slight measurement differences. We do not believe that these potential differences could have changed the outcome of the present study.

## Conclusion

The newly developed MND-DS may be a valuable tool for remotely monitoring respiratory function between clinic visits, as of all symptoms, it is the most accurate in identifying whether patients with MND have reduced respiratory function. The MND-DS showed better diagnostic performance than the ALSFRS-R respiratory domain, suggesting that the use of the MND-DS is preferred in the attempt to identify patients with a reduced respiratory function. Symptoms related to sleep quality or sleepiness/fatigue did not appear to be useful for screening reduced respiratory function. To further improve remote monitoring of respiratory function, the assessment of the MND-DS could be combined with home-based VC testing. Future research should evaluate the feasibility of home-based VC testing in patients with MND and re-address the respiratory domain of the ALSFRS-R.

## Electronic supplementary material

Below is the link to the electronic supplementary material.

Supplementary file1 (PDF 186 kb)

Supplementary file2 (PDF 150 kb)
