# Supplementary material for: Using patient-reported symptoms of dyspnea for screening reduced respiratory function in patients with motor neuron diseases
Source: J Neurol. 2020 Jun 23;267(11):3310–8. doi: 10.1007/s00415-020-10003-5 (PMC7578163; doi:10.1007/s00415-020-10003-5)
Supplement: Supplementary file 1 — Supplementary file1 (PDF 186 kb) [file 415_2020_10003_MOESM1_ESM.pdf]

Hypoventilation Symptom Questionnaire (HYSQ)

Please indicate the extent to which you experienced the following symptoms in the last 2 weeks:

1. At night I wake up often.

Not at all	**0** O	**1** O	**2** O	**3** O	**4** O	To a great extent

2. When I wake up at night, it takes a long time before I fall asleep again.

Not at all	**0** O	**1** O	**2** O	**3** O	**4** O	To a great extent

3. At night I have nightmares.

Not at all	**0** O	**1** O	**2** O	**3** O	**4** O	To a great extent

4. I wake up at night/in the morning drenched in sweat.

Not at all	**0** O	**1** O	**2** O	**3** O	**4** O	To a great extent

5. I feel tired when I wake up in the morning.

Not at all	**0** O	**1** O	**2** O	**3** O	**4** O	To a great extent

6. I experience headaches after I wake up in the morning.

Not at all	**0** O	**1** O	**2** O	**3** O	**4** O	To a great extent

7. I find it difficult to stay awake during the day (e.g. while watching TV or reading a book).

Not at all	**0** O	**1** O	**2** O	**3** O	**4** O	To a great extent

8. I experience fatigue during the day.

Not at all	0	1	2	3	4	To a great extent
	O	O	O	O	O	

9. I have difficulties concentrating (e.g. when watching TV or reading a book).

Not at all	0	1	2	3	4	To a great extent
	O	O	O	O	O	

10. I suffer from pneumonia (i.e. excessive coughing and mucus in my throat).

Not at all	0	1	2	3	4	To a great extent
	O	O	O	O	O	

11. I feel short of breath when sitting still.

Not at all	0	1	2	3	4	To a great extent
	O	O	O	O	O	

12. I feel short of breath when talking or eating.

Not at all	0	1	2	3	4	To a great extent
	O	O	O	O	O	

13. I feel short of breath when I lie flat on my back.

Not at all	0	1	2	3	4	To a great extent
	O	O	O	O	O	

14. I feel short of breath during light activities (e.g. walking, washing or getting dressed).

Not at all	0	1	2	3	4	To a great extent
	O	O	O	O	O	
