# Supplementary material for: Using patient-reported symptoms of dyspnea for screening reduced respiratory function in patients with motor neuron diseases
Source: J Neurol. 2020 Jun 23;267(11):3310–8. doi: 10.1007/s00415-020-10003-5 (PMC7578163; doi:10.1007/s00415-020-10003-5)
Supplement: Supplementary file 2 — Supplementary file2 (PDF 150 kb) [file 415_2020_10003_MOESM2_ESM.pdf]

Motor Neuron Disease – Dyspnea Scale (MND-DS)

Please indicate the extent to which you experienced the following symptoms in the last 2 weeks:

1. I feel short of breath when talking or eating.

	0	1	2	3	4	
Not at all	O	O	O	O	O	To a great extent

2. I feel short of breath when I lie flat on my back.

	0	1	2	3	4	
Not at all	O	O	O	O	O	To a great extent

3. I feel short of breath during light activities (e.g. walking, washing or getting dressed).

	0	1	2	3	4	
Not at all	O	O	O	O	O	To a great extent
